# Deciphering resistance mechanisms and novel strategies to overcome drug resistance in ovarian cancer: a comprehensive review

**DOI:** 10.32604/or.2024.031006

**Published:** 2024-04-23

**Authors:** EFFAT ALEMZADEH, LEILA ALLAHQOLI, AFROOZ MAZIDIMORADI, ESMAT ALEMZADEH, FAHIMEH GHASEMI, HAMID SALEHINIYA, IBRAHIM ALKATOUT

**Affiliations:** 1 Infectious Diseases Research Center, Birjand University of Medical Sciences, Birjand, Iran; 2 Department of Midwifery, Ministry of Health and Medical Education, Tehran, Iran; 3 Student Research Committee, Shiraz University of Medical Sciences, Shiraz, Iran; 4 Department of Biotechnology, Faculty of Medicine, Birjand University of Medical Sciences, Birjand, Iran; 5 Cellular and Molecular Research Center, Birjand University of Medical Sciences, Birjand, Iran; 6 Social Determinants of Health Research Center, Birjand University of Medical Sciences, Birjand, Iran; 7 Kiel School of Gynaecological Endoscopy, Campus Kiel, University Hospitals Schleswig-Holstein, Kiel, Germany

**Keywords:** Chemotherapy, Drug resistance mechanisms, Ovarian cancer, PARP inhibitors, VEGF inhibitor

## Abstract

Ovarian cancer is among the most lethal gynecological cancers, primarily due to the lack of specific symptoms leading to an advanced-stage diagnosis and resistance to chemotherapy. Drug resistance (DR) poses the most significant challenge in treating patients with existing drugs. The Food and Drug Administration (FDA) has recently approved three new therapeutic drugs, including two poly (ADP-ribose) polymerase (PARP) inhibitors (olaparib and niraparib) and one vascular endothelial growth factor (VEGF) inhibitor (bevacizumab) for maintenance therapy. However, resistance to these new drugs has emerged. Therefore, understanding the mechanisms of DR and exploring new approaches to overcome them is crucial for effective management. In this review, we summarize the major molecular mechanisms of DR and discuss novel strategies to combat DR.

## Introduction

Ovarian cancer (OC) is an endocrine-related cancer [1], ranking as one of the most common and deadliest gynecological cancers worldwide, with 313,959 new cases and 207,252 deaths in 2020 [2–4]. Despite advancements in various cancer treatment modalities, including surgical techniques, chemotherapy, radiotherapy, targeted therapies, and hormone therapy [5], OC remains a leading cause of death among gynecological cancers. Furthermore, OC has a high recurrence rate, with the mean time to recurrence being approximately 2 years. Post-relapse treatments are more aggressive, resulting in increased toxicity, resistance to chemotherapeutic drugs, and substantial financial burdens on patients, along with a diminished quality of life [5]. One of the primary reasons for the poor prognosis of OC is drug resistance (DR), particularly to platinum-based compounds. Around one-third of patients do not respond to initial platinum-based chemotherapy, and over time, 80% of other patients develop chemotherapy resistance, rendering disease recurrence practically incurable [6].

DR is characterized by cancer cells’ ability to survive exposure to anticancer drugs [7]. It occurs when diseases become tolerant to pharmaceutical agents [8]. DR results from various factors that pose a significant challenge in practical medicine, leading to cellular tolerance and ineffectiveness against one or more pharmaceutical agents [9]. Chemoresistant OC can be attributed to overexpressed proteins and alterations in signaling pathways. Genes associated with DR affect cellular processes, such as drug efflux, apoptosis, and DNA damage and repair. Additionally, cancer stem cells (CSCs) within ovarian tumor tissue contribute to chemoresistance [10].

The prognosis and therapeutic recommendations for OC depend on various prognostic factors, including patient age at diagnosis, performance status, and disease stage [3]. Several biological mechanisms impact the prognosis of OC, with aging playing a significant role. These mechanisms include alterations in cellular and humoral immunity, inflammation, inflammaging factors like interleukin 6 and C-reactive protein, vascular endothelial growth factor (VEGF) and its modulation, cell cycle control, methylation changes, low Vitamin D levels, the insulin/IGF-1 pathway, and oxidative stress [11]. The treatment of OC involves a multidisciplinary approach, including cytoreductive surgery to remove tumors, systemic chemotherapy, and, on rare occasions, radiotherapy [3]. Currently, five treatment options are considered standard for late-stage OC:

1) Cytoreducing surgery followed by platinum/taxane intravenous chemotherapy; 2) Cytoreducer surgery with intraperitoneal/intravenous chemotherapy; 3) Cytoreducing surgery followed by platinum/taxane-based intravenous chemotherapy in combination with bevacizumab and bevacizumab; 4) Neoadjuvant chemotherapy (NACT) with cytoreductive surgery performed between cycles 3 and 6, followed by chemotherapy; 5) Chemotherapy for patients who are not suitable for surgery or who progress during NACT [3]. Recent years have seen the emergence of potential therapeutic targets for OC, including anti-VEGF/VEGF receptor angiogenic inhibitors, non-VEGF angiogenic inhibitors, poly (ADP-ribose) polymerase (PARP) inhibitors, epidermal growth factor receptor (EGFR) inhibitors, folate receptor inhibitors, and epidermal growth factor receptor (EGFR) inhibitors [12]. Targeted therapies like bevacizumab and PARP inhibitors have become part of the standard first-line treatment for OC [13]. These therapies have improved progression-free survival (PFS) when administered concurrently with chemotherapy and/or maintenance therapy [13].

Despite initial responses to treatment, most women diagnosed with high-grade serum OC (HGSC) experience recurrent disease and chemotherapy resistance. The 5-year survival rate for women with HGSC ranges from 35% to 40%, primarily due to primary treatment resistance in 15%–25% of patients and the development of chemotherapy resistance in the majority of the remaining cases [14]. Therefore, this review aims to explore the use of chemical drugs in OC treatment, the mechanisms of DR, and novel strategies to overcome DR in OC.

## Ovarian Cancer: Pathogenesis, Histological Heterogeneity, and Progression

As of now, no widely accepted pathogenesis has been established for OC [15]. One of the significant challenges lies in the fact that OC is not a single disease; rather, it comprises a diverse group of tumors that can be categorized based on distinct morphological and molecular genetic characteristics [16,17]. Ovarian carcinoma may originate from any of three potential sites: the ovarian surface, fallopian tube, or the peritoneal cavity lined with mesothelium [18]. The tumorigenesis of ovarian carcinoma then progresses through a sequential mutation process, leading from a slow-growing borderline tumor to either a well-differentiated carcinoma (Type I) or a genetically unstable high-grade serous carcinoma that metastasizes rapidly (Type II) [18,19]. During the initial stages of tumorigenesis, the cells of ovarian carcinoma undergo an epithelial-mesenchymal transition, involving changes in the expression of cadherin and integrin, along with an upregulation of proteolytic pathways [18,20]. Carried by peritoneal fluid, cancerous cell spheroids evade anoikis and preferentially attach to the abdominal peritoneum or omentum, where they revert to their epithelial phenotype [18]. Early metastatic stages are regulated through controlled interactions of adhesion receptors and proteases, while late metastasis is marked by rapid oncogenic growth of tumor nodules on mesothelium-covered surfaces, resulting in ascites, intestinal obstruction, and tumor cachexia [18]. Approximately 90 out of every 100 ovarian tumors originate from epithelial cells [21]. The most common types of ovarian carcinoma based on histology are serous, clear-cell, endometrial, and mucinous tumors, all falling under the epithelial category [22]. Surface epithelial ovarian carcinoma is further divided into two subtypes: Type 1 and Type 2 tumors [16,17]. Type I tumors consist of low-grade serous carcinomas, low-grade endometrial carcinomas, clear cell carcinomas, mucinous carcinomas, and transient cell (Brenner) tumors [22]. Common genetic alterations in Type I tumors include Kirsten Rat Sarcoma Viral Oncogene Homolog (KRAS), V-Raf murine sarcoma viral oncogene homolog B1 (BRAF), Phosphatase and tensin homolog (PTEN), phosphatidylinositol-4,5-bisphosphate 3-kinase, catalytic subunit alpha (PIK3CA), Catenin Beta 1 (CTNNB1), and AT-rich interactive domain-containing protein 1A (ARID1A) [23]. Type II is more lethal and is primarily linked to continuous ovarian cycles, leading to inflammation and endometriosis [16] and is characterized by mutations in the tumor protein p53 (TP53) gene [23].

Numerous genetic and epigenetic changes are responsible for the transformation of ovarian carcinoma cells [18,19]. OC can be categorized into low-grade and high-grade tumors based on genetic alterations. Low-grade tumors often exhibit mutations in KRAS, BRAF, and PIK3CA, loss of heterozygosity (LOH) on chromosome Xq, microsatellite instability, and expression of amphiregulin. High-grade tumors, on the other hand, are associated with TP53 aberrations and potential BRCA1 and BRCA2 aberrations, as well as LOH on chromosomes 7q and 9p [24]. It has also been demonstrated that there is a 3.6% increase in OC mortality, which is positively linked to the Environmental Quality Index (EQI) comprising five domains: air, water, land, built environment, and sociodemographic factors [25].

The most commonly used biomarker for detecting OC is cancer antigen 125 (CA125), but its limited specificity constrains its utility. To enhance diagnostic accuracy, recent developments include the creation of multimarker panels that combine molecular biomarkers such as human epididymis secretory protein 4 (HE4), ultrasound findings, and menopausal status. Clinical applications of risk assessment tools like the Risk of Ovarian Malignancy Algorithm (ROMA), the Risk of Malignancy Index (RMI), and OVA1 tests have shown improved sensitivity and specificity. Ongoing research efforts are exploring novel biomarkers like autoantibodies, circulating tumor DNA (ctDNA), microRNAs (miRNAs), and DNA methylation signatures to provide early detection methods for OC [26].

Tumor heterogeneity in OC may arise from different anatomical sites [27], and this is a primary contributor to therapeutic failures and resistance to cancer treatments [28]. Key determinants of pharmacoresistance in OC include pharmacological parameters [29], molecular mechanisms [30,31], CSCs [32], and agents within the tumor microenvironment (TME) [33].

CSCs have been identified as crucial tumor-initiating factors that play a significant role in tumor recurrence after chemotherapy, employing various mechanisms to resist chemotherapy [34]. These mechanisms include ATP-binding cassette transporters, aldehyde dehydrogenase, DNA repair, and signaling pathways [32].

DR in OC is attributed to a variety of molecular mechanisms [30], which may relate to the development of resistance to chemotherapeutic agents, including alterations in drug transport, changes in cellular proteins involved in detoxification, modified drug targets, shifts in DNA repair mechanisms, and increased tolerance to drug-induced DNA damage [30,31].

Non-coding RNAs, including long non-coding RNAs, microRNAs, and circular RNAs, have been implicated in the development of DR in OC. Aberrantly expressed non-coding RNAs can promote resistance to OC by inhibiting apoptosis, inducing protective autophagy, enhancing abnormal tumor cell proliferation, driving epithelial-mesenchymal transition, promoting abnormal glycolysis, facilitating drug efflux, and restricting the apoptosis of cancer cells [35].

Recent research highlights the significant role of the TME in OC tumorigenesis [33]. The TME encompasses the tumor’s vasculature, connective tissue, infiltrating immune cells, and the extracellular matrix (ECM) [36].

## Mechanisms of DR in OC

**Figure 1:**
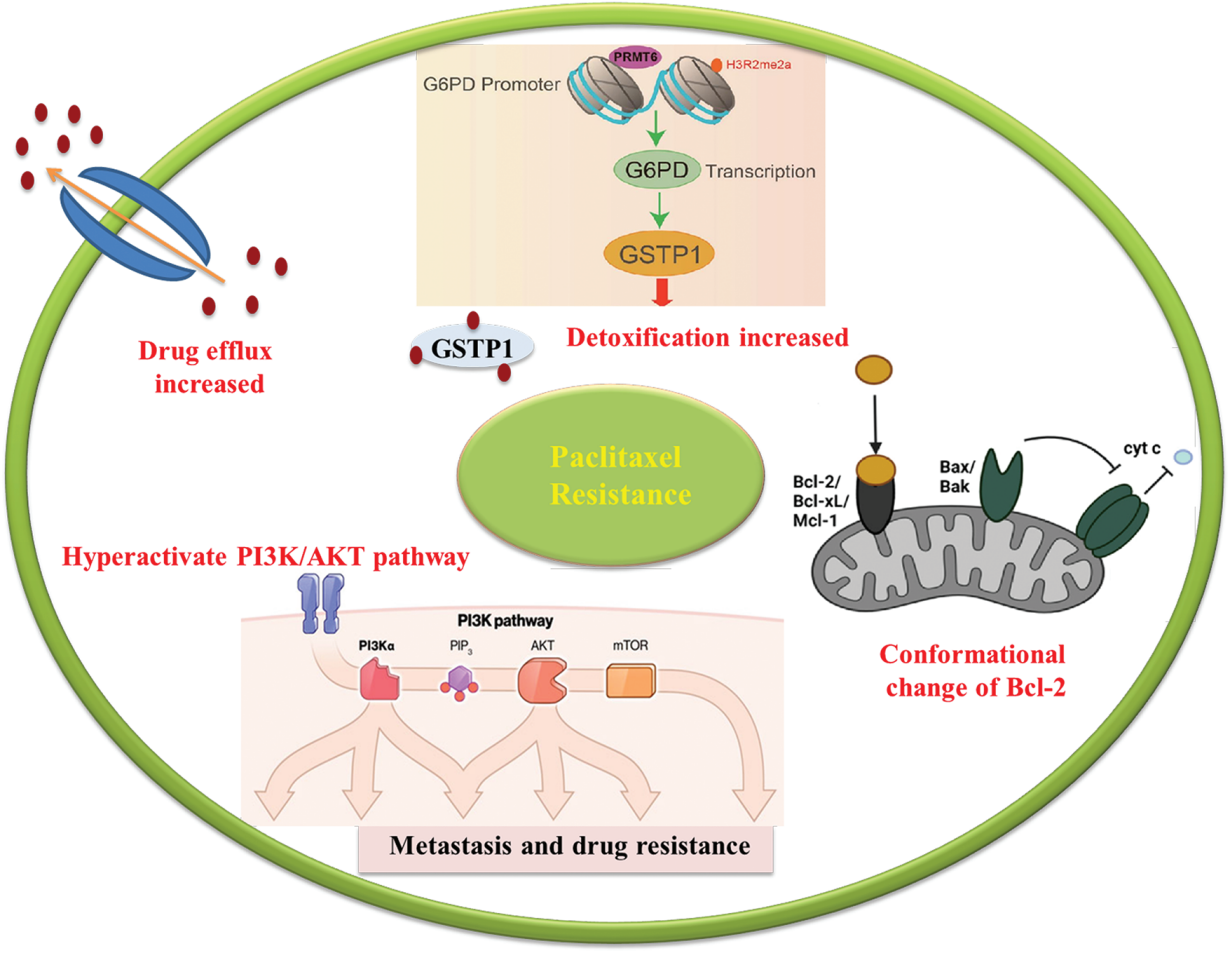
Schematic illustration of resistance mechanism of platinum. Resistance to platinum-based drugs has been associated to several mechanisms including influx and efflux transporters, DNA damage repair (DDR), Apoptotic pathways and Autophagy.

**Figure 2:**
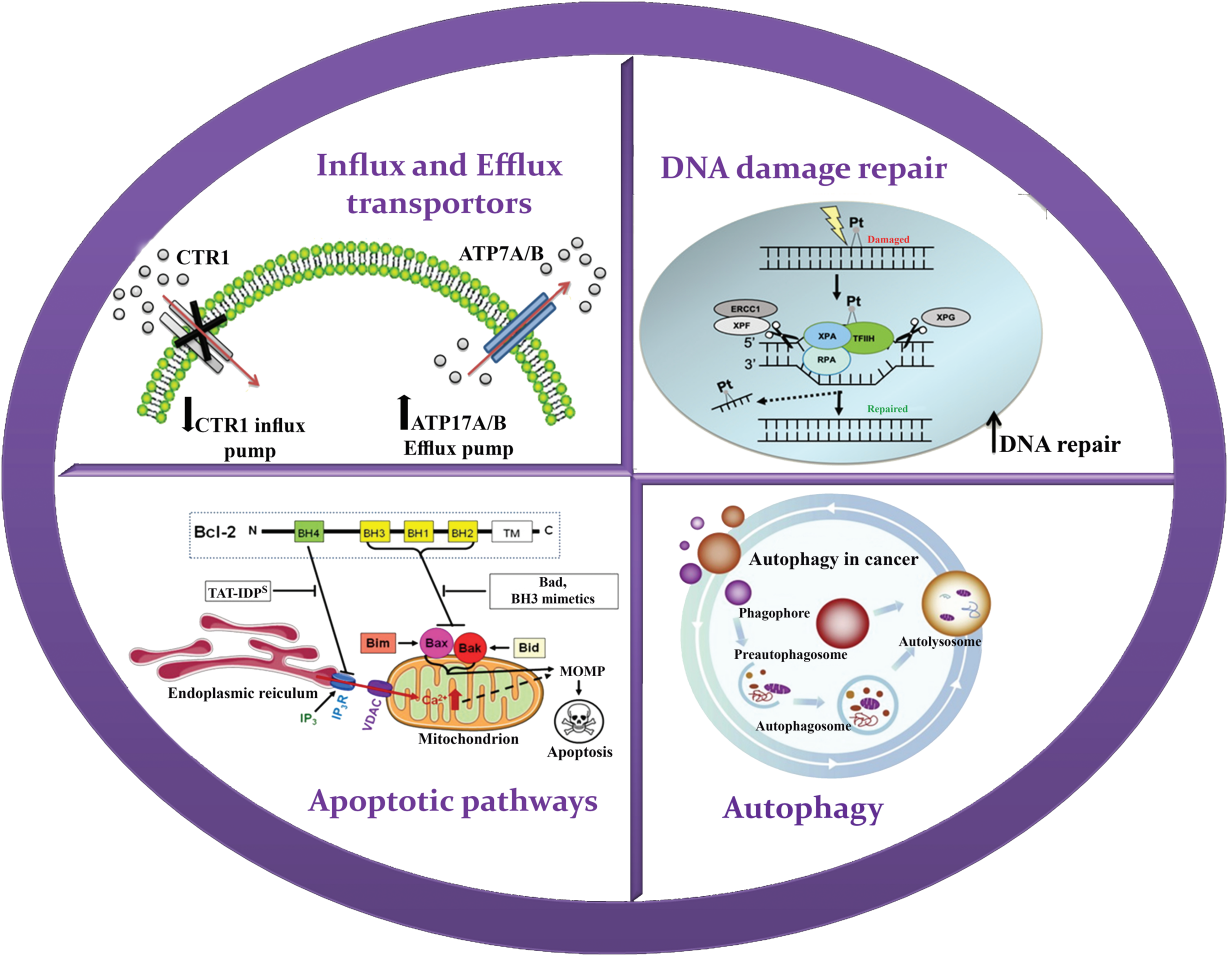
Schematic illustration of resistance mechanism of paclitaxel. Resistance to paclitaxel including efflux by P-glycoprotein, Phosphoinositide 3-kinase/protein kinase B pathway, glutathione S transferase 1 and B-cell lymphoma 2 family.

**Figure 3:**
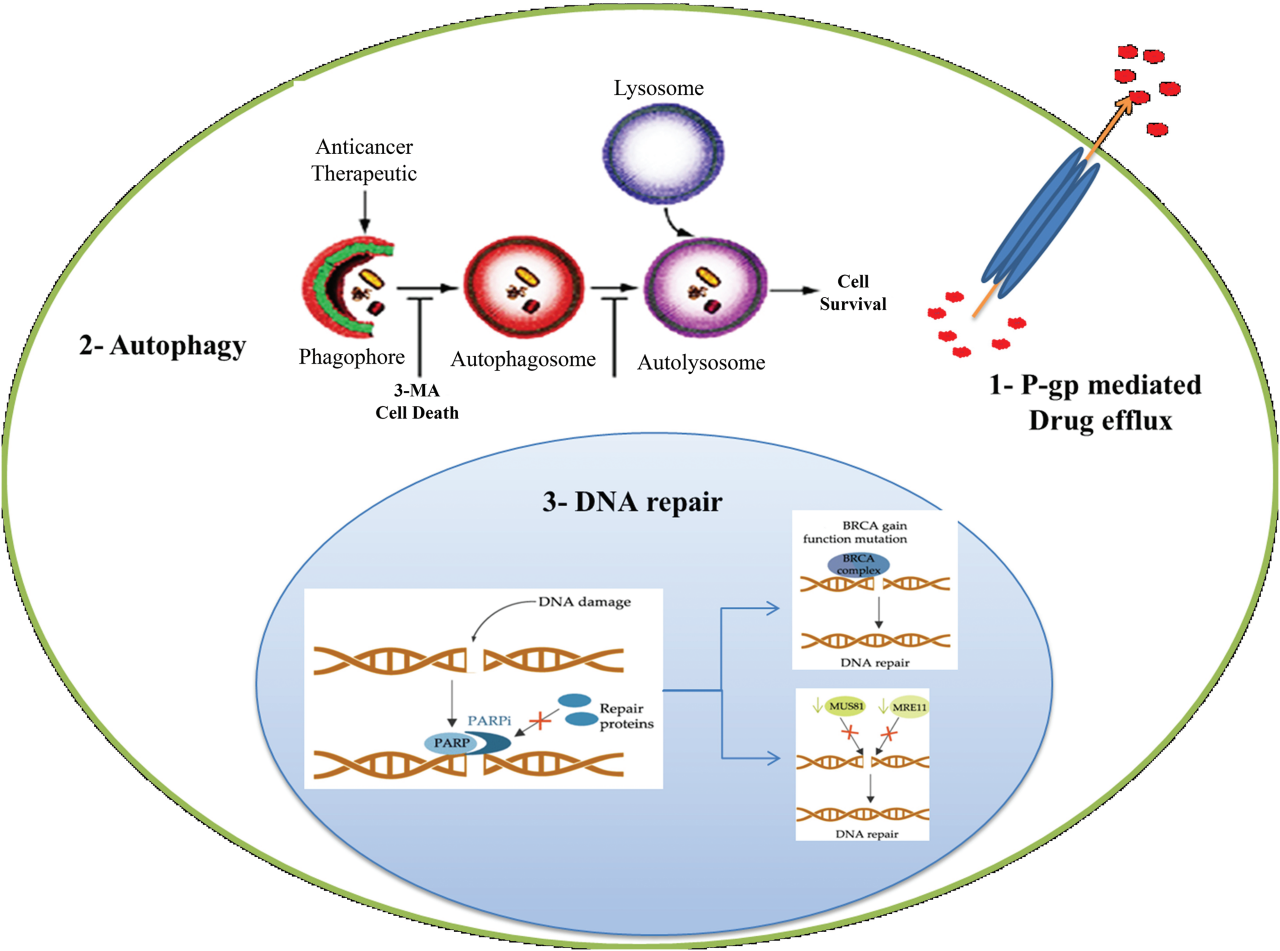
Schematic illustration of resistance mechanism of PARP inhibitor. Resistance to paclitaxel including DNA repair, Efflux transporters and Autophagy.

DR is a significant concern in medical practice as it leads to reduced cell sensitivity and failure to respond to one or more drugs. Initially, DR was observed in bacterial infections that were resistant to antibiotics, but later it was identified in various human disorders, including cancer [37]. DR is a major contributor to high mortality rates in cancer, and addressing it remains an urgent necessity [38,39]. Studies conducted over the past few decades have revealed that cancer cells possess distinct metabolic pathways compared to healthy cells. These pathways are responsible for their resistance to various chemotherapeutic drugs [40]. A cell’s metabolic pathways include a network of interacting genes, proteins, and metabolite reactions, all of which are regulated by structures like proteins and signaling molecules [41]. In cancer cells, many of these regulatory networks are dysregulated, leading to uncontrolled growth and proliferation [41]. Besides altered metabolic and signaling pathways, changes in the expression and activity of drug-metabolizing enzymes also play a vital role in DR [42]. Several mechanisms contribute to the development of DR, depending on the drug and cancer tumor [43]. Therefore, understanding the various mechanisms of resistance is crucial for planning effective therapies and improving outcomes for cancer patients (Figs. 1–3). In this review, we will delve into the mechanisms of resistance that occur with conventional chemotherapeutic drugs approved by the Food and Drug Administration (FDA) for the treatment of OC.

### Influx and efflux transporters

Platinum resistance in OC is caused by the dysregulation of influx and efflux pumps, such as copper transporter 1 (CTR1), ATPase copper-transporting alpha (ATP7A), and ATPase copper-transporting beta (ATP7B), which affect the transport of cisplatin. Cells sensitive to cisplatin express higher levels of CTR1, allowing for increased cisplatin influx. In contrast, overexpression of ATP7A/B in cisplatin-resistant cells leads to the efflux of platinum. Clinical investigations have provided evidence supporting the predictive significance of ATP7B expression levels in individuals with ovarian and endometrial cancer undergoing cisplatin chemotherapy. The negative feedback loop caused by rapid CTR1 downregulation following cisplatin exposure is suggested to contribute to cisplatin resistance [44,45]. In the case of paclitaxel, overexpression of efflux pumps, such as P-gp, can reduce its intracellular concentration and decrease its effectiveness. Recent research findings have indicated that mutations occurring in P-gp can inhibit the efflux process and result in a shift from efflux to influx pumps. Drug efflux pumps, such as ABCB1, also contribute to OC resistance to PARP inhibition by actively effluxing drugs from cells [46–48].

### DNA damage repair (DDR)

Once platinum drugs are transported into the cytoplasm through the CTR1 protein, they subsequently enter the nucleus, where they interact with DNA to create both intrastrand and interstrand crosslinks. The DNA lesions induced by platinum drugs, such as cisplatin, can lead to cell death through apoptosis [44]. Resistance to platinum compounds can be induced through DNA damage repair pathways or by ignoring the damage that has occurred. Double-strand DNA damage is typically repaired through two main pathways: homologous recombination and non-homologous end-joining (NHEJ). These mechanisms are responsible for restoring the integrity of the DNA molecule. On the other hand, single-strand lesions are primarily repaired through a process called nucleotide excision repair (NER), which helps correct any abnormalities in the DNA structure [49]. In the NER pathway, specific endonucleases such as excision repair cross-complementation group 1 (ERCC1)-XPF and XPG cleave the site of damaged nucleotides, followed by DNA synthesis to restore genetic integrity [50]. Higher levels of ERCC1 expression tend to be associated with platinum resistance in epithelial OC [51,44]. Early studies in several preclinical models suggested a correlation between NER proficiency and cisplatin resistance [52]. Tumor suppressors BRCA1 and BRCA2, which encode proteins involved in DDR, are important components of the homologous recombination mechanism. Mutations in BRCA1/2 are associated with high sensitivity to DNA-damaging agents and an enhanced overall response to platinum therapy. In other words, the restoration of tumor suppressor BRCA activity can increase resistance in OC [51,53]. Also, resistance to PARP inhibition is commonly caused by DNA repair activity, and patients with impaired homologous recombination pathways respond better to PARP inhibition treatment. BRCA1/2 secondary mutations can enhance the microhomology-mediated end-joining (MMEJ) pathway, allowing OC cells to overcome PARP inhibition [54–56].

### Apoptotic pathways

The effectiveness of chemotherapy heavily relies on the capacity of OC cells to undergo apoptosis triggered by drug treatment. The activity of the apoptotic pathways is controlled by the balance of proapoptotic and antiapoptotic proteins of the B-cell lymphoma 2 (BCL2) family [57]. Alterations in the expression levels of these proteins can exert a substantial influence on chemotherapy response and contribute to the development of DR. In the context of OC cells, an increase in the expression of anti-apoptotic proteins or a decrease in proapoptotic proteins can grant resistance to the commonly employed chemotherapy drug, cisplatin. Anti-apoptotic proteins like BCL2 and BCL-XL counteract apoptotic signals, favoring cell survival. Elevated expression of these proteins can impede the initiation of downstream apoptotic pathways, diminishing the effectiveness of cisplatin treatment. Conversely, diminished expression or compromised function of proapoptotic proteins, such as BAX and BAK, can also play a role in cisplatin resistance. These proapoptotic proteins promote apoptosis by facilitating the release of cytochrome c from the mitochondria and the activation of caspases, which are pivotal executors of the apoptosis process. When their levels decrease or their functions are impaired, the apoptotic response to cisplatin is hindered, enabling OC cells to survive and continue their proliferation [58].

### Autophagy

Autophagy is a cellular process involved in the degradation and recycling of damaged or unnecessary cellular components, including proteins, lipids, and organelles. It has been suggested that autophagy may play a role in resistance to PARP inhibitors. PARP inhibitors work by blocking the activity of an enzyme called PARP, which is involved in repairing damaged DNA. In cancer cells with defects in other DNA repair pathways, such as those caused by mutations in the BRCA1 or BRCA2 genes, PARP inhibitors can lead to DNA damage accumulation and cell death. However, recent studies have suggested that cancer cells can develop resistance to PARP inhibitors through various mechanisms, such as the activation of autophagy. In particular, autophagy has been shown to promote the survival of cancer cells treated with PARP inhibitors by removing damaged proteins and organelles, thereby reducing the accumulation of toxic cellular debris and promoting cell survival [43,59].

Previous studies have also indicated that autophagy in cisplatin-resistant ovarian. At present, autophagy in cancer cells is considered a potential DR mechanism [60].

### Induction of reactive oxygen species

Reactive oxygen species (ROS) are highly reactive molecules that can inflict damage upon cells and tissues [61]. Chemotherapy drugs such as cisplatin and doxorubicin can trigger ROS production as part of their mechanism of action [62]. Nevertheless, prolonged and excessive ROS production can lead to chemoresistance [63]. This can transpire through the upregulation of antioxidant enzymes, which can scavenge ROS and diminish their harmful effects [64]. Additionally, ROS can activate signaling pathways that bolster cell survival and inhibit apoptosis, culminating in chemoresistance [65].

### Phosphoinositide 3-kinase/protein kinase B pathway

The Phosphoinositide 3-kinase/protein kinase B (PI3K/AKT) pathway holds substantial significance in metastasis and DR across a range of cancer types, including OC. In OC, alterations in key elements of this pathway play a role in activating pro-survival signals and fostering resistance to chemotherapy, particularly drugs like paclitaxel. One crucial regulator of the PI3K/AKT pathway is the phosphatase and tensin homolog (PTEN), which functions as a negative regulator by inhibiting PI3K activity and dampening downstream AKT signaling. However, OC frequently exhibits a loss of PTEN function, which can transpire through various mechanisms such as genetic mutations or epigenetic changes, resulting in decreased PTEN expression or complete absence of PTEN protein. The absence of PTEN function leads to hyperactivation of the PI3K/AKT pathway, causing enhanced cell survival, proliferation, and resistance to chemotherapy. Moreover, mutations in the PI3K gene itself can also contribute to pathway dysregulation in OC. These mutations can sustain PI3K activation, even in the absence of growth factors or other stimuli. This sustained activation fosters cell survival, proliferation, and resistance to chemotherapy agents like paclitaxel. Another common alteration observed in OC is the hyperactivation of AKT, a downstream effector of the PI3K pathway. This hyperactivation can result from both PTEN loss and PI3K mutations. Activated AKT promotes cell survival and proliferation by phosphorylating and deactivating various downstream targets that participate in cell cycle regulation and apoptosis. When paclitaxel is administered, the excessive activation of the PI3K/AKT pathway suppresses the drug’s anti-proliferative signals, thus contributing to resistance and treatment failure [66].

### Glutathione S-transferase 1

In healthy cells, increased activity of protein arginine methyltransferase 6 (PRMT6) inhibits the formation of the precursor of glutathione S-transferase GSTP1. However, in the case of cancer cells, the downregulation of PRMT6 leads to an increased production of GSTP1. When cancer cells are exposed to paclitaxel, GSTP1 plays a crucial role in DR by capturing and detoxifying paclitaxel within the cells. This capturing process prevents paclitaxel from binding to its intended target, tubulin, which is essential for its anti-cancer effects. By efficiently sequestering paclitaxel, GSTP1 decreases the concentration of the drug available for binding to tubulin, thus disrupting microtubule dynamics. This mode of action results in reduced sensitivity of cancer cells to the cytotoxic effects of paclitaxel, contributing to the development of DR [67].

### B-cell lymphoma 2 family

The Bcl-2 family encompasses pro-apoptotic factors such as Bcl-2–associated death promoter and Bcl-2–associated X protein, as well as anti-apoptotic factors, including Bcl-2, B-cell lymphoma-extra-large, and myeloid leukemia 1 [68]. Some studies have proposed that paclitaxel can convert Bcl-2 into a pro-apoptotic factor by triggering the release of cytochrome c from the mitochondria [69]. However, the upregulation of anti-apoptotic members of the Bcl-2 family is also associated with resistance to paclitaxel, as these proteins hinder the production of Fas ligand (FasL), a ligand involved in cell death, by inhibiting its gene transcription [44,68].

### Alterations in tubulin

Paclitaxel, a widely employed chemotherapy medication, exerts its anti-cancer properties through its interaction with tubulin, a pivotal protein crucial for cell division. Nevertheless, modifications in tubulin can markedly influence the interaction with paclitaxel, potentially compromising its capacity to interfere with microtubule formation. Mutations in the genes responsible for tubulin can lead to substitutions of amino acids within paclitaxel’s binding site or alter tubulin’s overall structure. Additionally, post-translational adjustments like acetylation, phosphorylation, or glycosylation can also impact how paclitaxel binds to tubulin. These alterations can take place at specific amino acid positions in tubulin, affecting the accessibility or shape of paclitaxel’s binding site. Consequently, changes in the tubulin structure might reduce paclitaxel’s affinity for binding, which, in turn, limits its ability to disrupt microtubule formation and hinder the division of cancer cells [69,70].

### Alterations in drug metabolism

Paclitaxel, a widely employed chemotherapy medication, undergoes transformations within the liver and is eventually removed from the body through bile. Modifications in drug metabolism can have a profound impact on how paclitaxel behaves within the body, potentially affecting its effectiveness in treating cancer. The liver plays a vital role in processing paclitaxel through various enzymatic reactions. Among these, cytochrome P450 3A4 (CYP3A4) is a crucial enzyme responsible for converting paclitaxel into its active forms that exhibit anti-cancer properties. Alterations in the function or production of CYP3A4 can influence how paclitaxel is metabolized. For example, genetic variations or interactions with other drugs that impact CYP3A4’s activity can alter the rate at which paclitaxel is processed. Impaired metabolism of paclitaxel can result in changes in the drug’s levels in the body, potentially diminishing its therapeutic effectiveness. Additionally, variations in other enzymes and transporters involved in paclitaxel metabolism can also contribute to changes in how the drug behaves in the body. These variations in enzyme and transporter function can arise from genetic factors, drug interactions, or underlying health conditions, all of which can influence how paclitaxel is absorbed, distributed, metabolized, and excreted, ultimately affecting its therapeutic response [71,72].

### Ephrin type-B receptor 4

Ephrin type-B receptor 4 (EphB4) is a tyrosine kinase receptor that plays a role in blood vascular morphogenesis and angiogenesis [73]. Li et al. demonstrated that EphB4 is overexpressed in bevacizumab-resistant OC. Ephrin type-B receptor 4 (EphB4) promotes angiogenesis by interacting with its ligand, ephrin-B2, expressed on endothelial cells, and promoting endothelial cell migration and proliferation, leading to the formation of new blood vessels within the tumor. This can contribute to tumor growth, metastasis, and resistance to anti-angiogenic therapies. EphB4 also activates downstream signalling pathways, such as PI3K/Akt and mitogen-activated protein kinase/extracellular signal-regulated protein kinase, which promote cell survival and proliferation. Additionally, EphB4 promotes tumor cell invasion by regulating cytoskeletal dynamics and cell motility [74,75].

### Alterations in endothelial cell function

The mechanism of DR is not well understood in this case, but it is probably related to alterations in endothelial cell function [76] and VEGF pathway signalling [77]. One possible cause of resistance to bevacizumab is the presence of different varieties of VEGF proteins in OC. A study by van der Bilt et al. showed that in OC we can find VEGF-A, VEGF-C and VEGF-D, which may be the reason for resistance of these changes to bevacizumab [78].

## New Approaches to Overcome DR

There is limited information available regarding the emergence of DR in OC [79]. In recent years, several alternative strategies for treating DR have emerged. In this section, we will explore these new approaches to overcome DR in OC.

### Combination therapy

Combination chemotherapy is a treatment approach utilized in OC to target cancer cells using two or more chemotherapeutic drugs. However, some cancer cells can develop resistance to this treatment. Resistance to standard combination chemotherapy in OC can occur through various mechanisms, such as altered drug metabolism, increased drug removal, the development of drug-resistant cell clones, a more aggressive cancer phenotype, immune evasion, and the creation of a hostile microenvironment [78,80]. For the past two decades, a combination of platinum and paclitaxel has been the accepted OC treatment. Recently, two PARP inhibitors (Olaparib and Niraparib) and a vascular endothelial growth factor inhibitor (Bevacizumab) have received FDA approval as maintenance treatments for OC [44]. As a result, novel approaches to combination therapy have emerged in recent years. One such approach involves the use of specific epigenetic drugs to delay the development of resistance [81]. Belinostat, a pan-histone deacetylase (HDAC) inhibitor, and decitabine, a DNA methyltransferase inhibitor, have demonstrated increased effectiveness in single-agent therapy in mice and *in vitro* [82]. A targeted treatment strategy that involves inhibiting HDAC6 in tumors with mutated AT-rich interaction domain 1A (ARID1A) has shown improved survival in xenografts. This success is attributed to the direct deacetylation of Lys120 by the cellular tumor antigen p53, inducing apoptosis in a specific cell type [83]. While combination chemotherapy may increase toxicity and side effects, it helps delay the emergence of resistance [84]. Therefore, alternative approaches to tackle this issue have emerged in recent years.

Approved drugs used in OC and their mechanism are presented in Table 1, while drug combinations used in OC are presented in Table 2.

**Table 1:** Approved drugs used in OC and their mechanism

Approved drug [85]	Mechanism of action
Alkeran (Melphalan)	Alkylates guanine and inhibits DNA and RNA synthesis and cytotoxicity [86].
Alymsys (Bevacizumab)	Binds to VEGF [87].
Avastin (Bevacizumab)	Selectively binding circulating VEGF, inhibiting the binding of VEGF to its cell surface receptors, reducing in microvascular growth of tumor blood vessels and limits the blood supply to tumor tissues [88].
Mvasi (Bevacizumab)	
Zirabev (Bevacizumab)	
Paraplatin (Carboplatin)	Forming reactive platinum complexes, causing the intra-and inter-strand cross-linkage of DNA molecules within the cell, modifying the DNA structure and inhibiting DNA synthesis [89].
Cisplatin	Crosslinking with the urine bases on the DNA, forming DNA adducts, preventing repair of the DNA leading to DNA damage and inducing apoptosis [90].
Cyclophosphamide	1) attachment of alkyl groups to DNA bases, and preventing DNA synthesis and RNA transcription from the affected DNA, 2) cross-linking bonds between atoms in the DNA and preventing synthesis or transcription of DNA, and 3) inducing of mispairing of the nucleotides leading to mutations [91,92].
Doxorubicin Hydrochloride	1) intercalating into DNA and disruption of topoisomerase-II-mediated DNA repair, 2) generating of free radicals and damage to cellular membranes, DNA and proteins [93].
Doxil (Doxorubicin Hydrochloride Liposome)	Decreasing the risk of cardiotoxicity by hanging tissue distribution and by decreasing the rate of drug release [94].
Elahere (Mirvetuximab soravtansine-gynx)	Binding to FRα, releasing the intracellular of DM4 via proteolytic cleavage, disrupting the microtubule network within the cell, arresting the cell cycle and apoptotic cell death [95].
Gemzar (Gemcitabine Hydrochloride)	Inhibit processes required for DNA synthesis by incorporation of dFdCTP into DNA and causes cell death [96].
Infugem (Gemcitabine Hydrochloride)	Uptaking into malignant cells, phosphorylating by deoxycytidine kinase, forming gemcitabine monophosphate, converting to dFdCDP and dFdCTP, Incorporating of dFdCTP into the DNA chain, and leading to chain termination, DNA fragmentation, and apoptotic cell death of malignant cells [97].
Hycamtin (Topotecan Hydrochloride)	Binding to the topoisomerase I-DNA complex, preventing relegation of these single-strand breaks, inducing replication arrest and lethal double-stranded breaks in DNA, leading to apoptosis (programmed cell death) [98].
Lynparza (Olaparib)	Acts as PARP inhibitor: acts on PARP1, PARP2, and PARP3, acts as a selective competitive inhibitor of NAD+ at the catalytic site of PARP1 and PARP2, inhibits the BER pathway, leads to the most toxic form of DNA damage by the accumulation of unrepaired SSBs, cause to apoptotic cell death [99,100].
Zejula (Niraparib Tosylate Monohydrate)	Inhibiting PARP enzymatic activity, increasing the formation of PARP-DNA complexes, damaging DNA, apoptosis, and cell death [101].
Niraparib Tosylate Monohydrate	
Paclitaxel	Binding to an apoptosis-stopping protein called Bcl-2 and inducing programmed cell death (apoptosis) [102].
Rubraca (Rucaparib Camsylate)	Inhibiting PARP1, PARP2, and PARP3.6, inhibiting PARP traps the enzyme on damaged DNA, halting the repair process and forming toxic PARP–DNA complexes, and by initiating DNA repair processes such as error-prone NHEJ or alternative end-joining pathways, leading to mutations or chromosomal change, damaging DNA and leading to cancer cell apoptosis and cell death [103].
Tepadina (Thiotepa)	Cross-linking guanine nucleobases in DNA double-helix strands, directly attacking DNA, preventing the cell from dividing, and stopping tumor growth [104].

Abbreviations: Bcl-2, B-cell leukemia 2; dFdCDP, Gemcitabine diphosphate; dFdCTP, Gemcitabine triphosphate; DNA, Deoxyribonucleic acid; FRα, Folate receptor alpha; NAD^+^, Nicotinamide adenine dinucleotide; NHEJ, Nonhomologous end joining; PARP, Poly-ADP ribose polymerase; RNA, Ribonucleic acid; SSBs, Single-strand breaks; VEGF, Vascular endothelial growth factor.

**Table 2:** Drug combinations used in OC [105]

Drug	Components
BEP:	B = Bleomycin
	E = Etoposide Phosphate
	P = Cisplatin (Platinol)
CARBOPLATIN-TAXOL:	Carboplatin
	Paclitaxel (Taxol)
GEMCITABINE-CISPLATIN:	Gemcitabine Hydrochloride
	Cisplatin
JEB:	J = Carboplatin (JM8)
	E = Etoposide Phosphate
	B = Bleomycin
PEB:	P = Cisplatin (Platinol)
	E = Etoposide Phosphate
	B = Bleomycin
VAC:	V = Vincristine Sulfate
	A = Dactinomycin (Actinomycin-D)
	C = Cyclophosphamide
VeIP:	Ve = Vinblastine Sulfate (Velban)
	I = Ifosfamide
	P = Cisplatin (Platinol)

### NSC23925

(2-(4-methoxyphenyl)-4-quinolinyl) (2-piper-idinyl) methanol (NSC23925), a compound that suppresses the expression of Pgp, has been shown to reduce the development of resistance to paclitaxel [106], Pgp is significantly overexpressed in all resistant cell lines [106]. When the ability of NSC23925 to reverse DR in multidrug-resistant (MDR) OC cell lines was evaluated, it was found to be 50 and 20 times more potent than verapamil and cyclosporin A (CsA), respectively [107]. Co-administration of NSC23925 with paclitaxel increases apoptosis, potentially preventing the development of paclitaxel resistance. The toxicity of NSC23925, as determined by blood cell count, body weight, and organ histology, is not significant whether NSC23925 is administered alone or in combination with paclitaxel [108].

### Ethacrynic acid

Ethacrynic acid (EA) is a FDA-approved specific inhibitor of GST, which has been shown to enhance the cytotoxicity of anticancer drugs and reverse DR [109]. Overexpression of GST is often associated with resistance to cytotoxic agents [110]. OC cells that developed resistance after exposure to low-dose melphalan for seven days or incrementally increased doses of melphalan for over a year showed elevated GST activity and mRNA levels compared to their parental cells [111]. Howeverthe seven-day melphalan-resistant cells quickly reverted to a drug-sensitive phenotype within two weeks of treatment withdrawal, indicating that the resistance was not stable. In contrast, the one-year treated cell line maintained a permanent resistant phenotype, even when melphalan treatment was discontinued. These results suggest a possible explanation for the improved efficacy of intermittent chemotherapy compared to continuous drug delivery in clinical settings [112]. Co-incubation with EA during the seven-day melphalan treatment in OC cells prevented the development of melphalan resistance, and this effect increased in a dose-dependent manner through a reduction in GST gene expression [111].

### Selenium compounds

The anticarcinogenic effects of selenium, a crucial dietary trace element, have been extensively studied [113]. Selenium-containing compounds, such as selenite and selenomethionine, have been shown to enhance the effectiveness of common cytotoxic drugs while reducing the adverse effects of chemotherapy [114]. Studies conducted on OC patients in both *in vitro* and *in vivo* settings have demonstrated that selenium compounds can delay the emergence of DR to melphalan, cisplatin, and carboplatin [115,116]. Notably, selenium-containing substances have been shown to prevent the amplification of the GST gene, a process that occurs during the development of melphalan resistance [116]. Selenium compounds can also reduce the levels of the antioxidant glutathione, which is known to increase after cisplatin treatment [117]. Treatment with cisplatin was more prolonged and effective when selenite compounds were added, preventing the growth of ovarian tumor xenografts [115]. These findings support the hypothesis that selenium compounds have genetic or epigenetic effects that hinder the development of DR [118].

### OC stem cells in multiDR

CSCs have the capacity to differentiate into any cell type within a tumor, self-replicate, and contribute to chemotherapy resistance. These characteristics play a significant role in the growth and recurrence of malignant tumors. Experimental studies suggest that dysregulation of ovarian CSCs may contribute to resistance to chemotherapeutic drugs [119]. One strategy to target CSCs is to inhibit signaling pathways [120]. In OC cells, platinum resistance can be overcome by knocking down β-catenin or using the Wnt-specific inhibitor iCG-001, which also reduces the number of stem cells [121]. Gamma-secretase inhibitors or siRNAs can render ovarian CSCs more sensitive to platinum. The combination therapy of GSI and cisplatin targets both CSCs and non-CSCs and has a more potent synergistic cytotoxic effect than cisplatin alone [122]. The anticancer effects are significantly enhanced when GSI and paclitaxel are combined in platinum-resistant xenografts [123].

### ncRNAs

There is growing evidence of an increasing number of aberrantly expressed microRNAs (miRs) in OC, and these miRs’ expression patterns have been linked to tumor subtypes, tumor stage, prognosis, and MDR [124]. In various preclinical studies, ncRNAs have been targeted to treat OC and reverse DR. Two fundamental miR-based treatment approaches have been employed to counter multiDR in OC: anti-miR therapy (antagomiRs) and miR replacement therapy (miR mimics). MiR mimics are short, synthetic double-stranded oligonucleotides chemically modified to resemble native miRs. Transfecting the miR-634 mimic into OC cells promotes apoptosis and resensitizes resistant cells to cisplatin [125]. AntagomiRs are anti-sense oligonucleotides (ASOs) that have undergone chemical modification to interfere with oncomiRs and hence disrupt miR-related pathways. AntagomiR (anti-miR-21 inhibitor transfection) decreases and increases PDCD4 and c-IAP2 expression, respectively, to revert the cisplatin-resistant phenotype in ovarian cell lines [124]. Technologies for lncRNA silencing, including ASOs and siRNA, have been used to overcome chemoresistance. When HOTAIR is destroyed, OC becomes more chemosensitive in living conditions [126].

### Autophagy

In the formation of MDR, autophagy, a survival mechanism utilized by organisms to handle various types of stress, has taken on a novel role [127]. Toxic elements can be removed via increased autophagy, potentially leading to a rise in MDR and enhanced cancer cell survival. Thus, suppressing autophagy [128] could have two possible effects: re-sensitizing cancer cells that are resistant to treatment and increasing tumor cell death. To stop autophagy, specific target-siRNA or inhibitors have been used. Increased cisplatin-induced cell death and drug resensitization in A2780CP cells are possible with berlin-1-targeted siRNA [129]. Autophagy inhibitors come in two different varieties: early-stage inhibitors, including Wortmannin, LY294002, and 3-MA [130]. And late-stage inhibitors, such as bafilomycin A1, chloroquine (CQ), and lysosomal protease inhibitors, which prevent autophagic cargo from being destroyed inside autolysosomes and the fusion of autophagosomes and lysosomes [131].

### Clonal evolution tumor heterogeneity

Recent studies suggest that tumor heterogeneity, driven by clonal evolution, may contribute to MDR in OC by promoting tumor sampling bias and phenotypic variance [28]. However, the mechanisms responsible for its emergence remain poorly understood. It has been proposed that genetically diverse clones may already exist within the tumor bulk prior to treatment. Subclones that acquire advantageous mutations can survive and proliferate. Chemotherapeutic drugs eliminate previously dominant sensitive clones, making way for resistant clones [132]. Furthermore, amplification of the AKT2 gene in OC is associated with paclitaxel resistance [133]. Results from a study involving 92 high-grade serous OC patients initially exhibiting refractory, resistant, sensitive, and acquired resistant disease demonstrated that mutations in tumor suppressor genes, including PTEN, retinoblastoma 1, neurofibromin 1, and RAD51B, lead to acquired chemotherapy resistance [132]. Various genes, the epigenetic silencing of which is hindered by heterogeneity, have also been linked to the development of platinum-based resistance in OC. These genes include armadillo repeat-containing X-linked 2, COL1A1, MDK, and mesoderm-specific transcript [134]. The application of cutting-edge treatments targeting specific molecular heterogeneities has shown promise in reversing DR (DR) in OC. In a phase I clinical trial involving high-grade epithelial OC patients unresponsive to platinum and taxanes, individuals with PTEN and PIK3CA mutations in the PI3K/AKT pathway appeared to benefit from the combination of the AKT inhibitor perifosine and docetaxel [135].

### CRISPR/Cas9

Genome editing allows for the modification of the genomes of various cell types and organisms. This innovative approach relies on the use of designed chimeric nucleases that incorporate non-specific DNA cleavage modules connected to DNA-binding domains with specific sequences (nuclease) [136]. Some of these tools include transcription-activator-like effector nucleases and zinc-finger nucleases [137]. Nevertheless, the CRISPR-Cas9 technique, which is now gaining widespread acceptance, has revolutionized the field of cancer modeling [138]. In a study, researchers investigated whether the ABCB1 gene could be silenced in the doxorubicin-sensitive, adriamycin-resistant (A2780/ADR) OC cell line using CRISPR/Cas9 genome editing technology. The findings of the study showed that the CRISPR/Cas9 system could significantly reduce P-gp expression. The abrupt reduction in ABCB1 gene expression correlated with increased doxorubicin sensitivity in cells transfected with sgRNAs. Using CRISPR-based approaches, the A2780/ADR cell line was successfully edited to revert to a nonmalignant phenotype, which proved effective in downregulating the target gene [139].

### Nanomedicine

Nanomedicine utilizes nanoscale materials, such as biocompatible nanoparticles [140] and nanorobots [141] for a variety of applications, including diagnosis [142], and drug delivery [143].

#### Nanoparticles

Nanoparticles deliver cancer drugs through two key mechanisms: they provide direct access to cells and serve as platforms for drug combinations. Nanotechnology applied to chemotherapy agents for cancer treatment overcomes DR by targeting various mechanisms [144]. Factors like size, properties, and the enhanced permeability and retention (EPR) effect are essential considerations in nanoparticle design [145]. In cancer therapy, nanoparticles with diameters of 10–100 nm can achieve EPR, effectively delivering drugs. However, particles smaller than 1–2 nm can escape from normal vasculature and harm healthy cells, while particles larger than 100 nm are eliminated from circulation by phagocytes [145]. Surface modifications can impact the bioavailability and half-life of nanoparticles. Making nanoparticles hydrophilic, for example, can extend their circulation times and enhance their penetration and accumulation in tumors [146]. Nanocarriers for drug delivery not only enhance the therapeutic efficiency of chemotherapy drugs but also protect normal cells from cytotoxicity. Nanoparticles can passively or actively target cells, effectively delivering chemotherapeutic agents [145,147]. Various types of nanoparticles are available for cancer treatment and addressing DR. We will discuss them in this section.

##### Liposomes

Liposomes, the most extensively studied nano-drug carriers for drug delivery, consist of spherical vesicles formed by one or more phospholipid bilayers. Liposomes offer significant advantages over traditional drug delivery systems, including targeted delivery, high biocompatibility, biodegradability, easy functionalization, low toxicity, prolonged drug release, and enhanced therapeutic efficacy. With the rapid development of nanotechnology, the exploration of liposome composition has become increasingly comprehensive. Varieties of liposome composition, including long-circulating PEGylated liposomes, ligand-functionalized liposomes, stimuli-responsive liposomes, and advanced cell membrane-coated biomimetic nanocarriers, impart unique physiological functions to drug delivery [148].

##### Polymeric and solid lipid nanoparticles

Polymeric nanoparticles stand out as essential tools for enhancing drug bioavailability and specific delivery at the target site. The versatility of polymers makes them suitable for tailoring drug delivery systems to specific requirements [149]. Drug-carrying polymer nanoparticles are created by attaching a copolymer to a polymer matrix. Nano formulations incorporate synthetic polymers and natural polymers like polycaprolactone (PCL) and poly (lactic-co-glycolic acid) [150]. Solid lipid nanoparticles (SLNs) are colloidal particles with sizes ranging from 50 to 1000 nm, composed of lipids, chemotherapeutic drugs, and surfactants. SLNs typically exhibit improved efficacy and the ability to combat DR by enhancing drug uptake in cancer cells and inducing apoptosis [151].

##### Micelles

Micelles are colloidal particles with sizes ranging from 5 to 100 nm and are currently under investigation as carriers for hydrophobic drugs in anticancer therapy [152]. Micelles facilitate the permeability and endocytosis of OC cells, preventing the targeting of normal cells OC cells [153]. They also mitigate DR through the EPR effect, active internalization, endosome-induced drug release, and evasion of water-insoluble chemotherapy drugs [154].

##### Dendrimers

Dendrimers are spherical nanoparticles with three-dimensional, multi-branched structures that can be engineered to have sizes ranging from 1 to 15 nm. These nanoparticles possess unique features, including a low polydispersity index, high water solubility, biocompatibility, multivalency, and high molecular weight. Due to these distinctive properties, dendrimers can encapsulate both hydrophilic and hydrophobic medications [151].

##### Mesoporous silica nanoparticles

Surface modifications investigated to facilitate drug loading show that mesoporous silica nanoparticles (MSNs) have high drug loading efficiency because of their high pore volume and surface area properties. In cancer cells, MSNs are capable of targeted and controlled drug delivery, leading to increased cellular absorption and delivery of therapeutic drugs at cellular levels [154]. It is accepted that MSNs are excellent drug delivery vehicles due to their enhanced pharmacokinetics and therapeutic efficacy [145].

##### RNA interference therapy

In studies, small interfering RNAs (siRNA), short hairpin RNAs, and antisense oligodeoxynucleotides have been proposed as therapeutic possibilities for the treatment of cancer [147]. siRNA-targeted MDR genes overcome DR by silencing P-gp or MDR1, MDR-associated protein 1, Bcl2, and breast cancer resistance protein. However, up to now, the therapeutic efficacy of these RNA interference strategies has been consistently unsatisfactory [151]. Therefore, encapsulating siRNAs in nanoparticles to prevent the rapid degradation of siRNA molecules leads to increased cellular targeting, improved effective absorption, and limits localization in normal cells [155].

##### Planetary ball-milled nanoparticles

Recently, there has been significant interest in planetary ball-milled nanoparticles. The main challenges in other drug delivery methods are poor aqueous solubility, limited bioavailability, and absorption [156]. However, planetary ball-milled nanoparticles are easy to manufacture. They consist of a starch core coated with biodegradable copolymers, resulting in a spherical shape and uniform particle size [147]. This easy production process is due to the starch core covered with biodegradable copolymers, creating a spherical shape with a uniform particle size. Furthermore, planetary ball-milled nanoparticles exhibit 100% loading efficiency for drugs, whether hydrophobic or hydrophilic, and offer control over logP levels for drug delivery [157]. In general, the round shape and particle size of less than 100 nm make planetary ball-milled nanoparticles highly effective for delivering drugs to tumor cells [158]. Their potential for drug encapsulation has led to the selective targeting of cancer cells, enhancing safety and efficiency [147].

##### Dual-action organoplatinum polymeric nanoparticles

A groundbreaking approach to combat drug-resistant organoplatinum polymeric nanoparticles (OPNPs) is the use of “dual-action” organometallic polymeric nanoparticles. These OPNPs are created by combining organoplatinum payloads with the anionic block copolymer methoxy polyethylene glycol-block-polyglutamic acid (MPEG5k-PGA50). The OPNPs improve the solubility and biocompatibility of hydrophobic organoplatinum payloads. These payloads are gradually released from the core of the OPNPs within the acidic environment of endosomes after entering cancer cells through endocytosis. In contrast to conventional platinum treatments, the organoplatinum compound operates in a “dual-action” mode, causing both mitochondrial and nuclear DNA damage. This dual-action approach makes drug-resistant OC cells susceptible to the organoplatinum payloads [159].

#### Cold responsive nanomaterial

A hybrid nanoparticle composed of phospholipids and polymers has been developed to overcome MDR in cancer stem cells using cold exposure. This cold-responsive hybrid HCLPN-D nanoparticle was designed for targeted delivery of chemotherapeutics (DOX) *in vitro* and in multidrug-resistant tumors *in vivo*. It is composed of hyaluronic acid (HA), chitosan, dipalmitoylphosphatidylcholine (DPPC), and poly (N-isopropyl acrylamide) (PNIPAM). When injected intravenously into the tail vein, the enhanced EPR effect of tumor vasculature facilitates the more effective delivery of drugs into tumors by HCLPN-D nanoparticles. Moreover, at low temperatures (12°C), these nanoparticles rapidly and permanently disintegrate, leading to the burst release of most of the enclosed medication. This combination of cold-triggered DOX burst release and low temperature effectively overcomes the resistance of NCI/RES-ADR cells to multiple drugs [160].

### The combination of gene and drug delivery

An additional strategy to enhance the success of oncotherapy involves combining gene and drug delivery. For example, HA-labeled poly(d,l-lactide-co-glycolide) nanoparticles loaded with focal adhesion kinase (FAK) siRNA and paclitaxel (PTX) were employed for OC treatment (HA-PLGA-NP-PTX+FAK siRNA). Tumor cells uptake more HA-PLGA-NP-PTX+FAK siRNA due to the presence of CD44, leading to reduced cell viability by inducing apoptosis in both SKOV3-TR and HeyA8-MDR cells. FAK siRNA effectively inhibits the AKT pathway, which is associated with metastasis and DR [161].

### Gas plasma

The introduction of gas plasma in oncotherapy as a novel therapeutic agent is expected to yield superior results compared to traditional medications. Gas plasma can also resensitize cancer cells that have become resistant to chemotherapy while sparing healthy cells. Current discussions emphasize the potential of combining cold atmospheric plasma (CAP), plasma-activated liquid (PAL), and traditional therapies such as chemotherapy, radiation therapy, pulsed electric fields, nanoparticles, and plant-based treatments to enhance efficacy. Recent research by Rasouli et al. has focused on the selective effect of gas plasma in oncotherapy and overcoming chemotherapy resistance [162]. The study used A2780 CP, SKOV-3, and granulosa cells as hypodiploid human cell lines, OC cell lines, and normal primary cells, respectively. The results showed that the selectivity indices of carboplatin and plasma-activated medium (PAM) for A2780 CP and SKOV-3 were significantly higher than those of traditional chemotherapeutic drugs. Particularly, PAM with 10% FBS exhibited the highest selectivity towards OC cells among all plasma treatment methods, surpassing the selectivity of various plasma therapies and conventional chemotherapeutic drugs [163].

### Metabolic approaches to overcoming chemoresistance in OC

Aerobic glycolysis and macromolecular synthesis are the primary metabolic alterations in cancer that hinder cancer cells from undergoing apoptosis. Many types of cancer display metabolic changes, such as increased glycolysis and mitochondrial dysfunction, which impede apoptosis and result in a constant demand for energy. Consequently, metabolic deprivation can significantly affect cancer cells. Given the high prevalence of chemoresistance and the rapid progression of OC, adopting metabolic strategies is a prudent approach to enhance the bleak prognosis associated with OC. The inhibition of glycolysis and other metabolic stressors leading to metabolic scarcity, along with the targeting of mitochondrial apoptotic machinery, appear to be effective strategies. For instance, a synergistic effect can be achieved in overcoming chemoresistance by combining anticancer chemotherapeutics with metabolic modulators. To gain a better understanding of the biomolecular mechanisms underlying the metabolic alterations and chemoresistance observed in OC, substantial basic and clinical research is still required. This will inspire researchers to develop novel drugs aimed at rectifying metabolic dysregulation, thus substantially improving OC management [135].

## Conclusion

The emergence of DR in OC presents a significant hurdle to effectively treating the disease, despite advancements in drug delivery and targeted therapies. Chemotherapy resistance in OC is a complex process influenced by multiple factors, underscoring the necessity for a deeper comprehension of the underlying molecular pathways to formulate innovative strategies for conquering DR.

This knowledge is particularly invaluable for custom-tailored therapies, which can anticipate the responses of cancer cells to existing chemotherapeutic medications and identify fresh therapeutic methods for managing OC. Novel approaches to combat resistance encompass combining therapies and employing personalized medicine, which may prove more efficacious than conventional single-agent therapies. In summary, an improved understanding of the molecular mechanisms behind DR in OC is essential for developing more efficacious treatments for this ailment.

## Data Availability

The datasets used and/or analysed during the current study are available from the corresponding authors on reasonable request.

## Abbreviation

OC: Ovarian cancer
DR: Drug resistance
VEGF: Vascular endothelial growth factor
HGSC: High-grade serum ovarian cancer
PARPi: Poly (ADP-ribose) polymerase inhibitors
TME: Tumor microenvironment
PLD: Pegylated liposomal doxorubicin
CR: Clinical response
DDR: DNA damage repair
CTR1: Copper transporter 1
HR: Homologous recombination
P-gp: P-glycoprotein
ROS: Reactive oxygen species
Bcl-2: B-cell lymphoma 2
MMEJ: Microhomology-mediated end joining
EphB4: Ephrin type-B receptor 4
MDR: Multidrug resistance
CSCs: Ovarian cancer stem cells
ASO: Anti-sense oligonucleotides
OPNPs: Organometallic polymeric nanoparticles
